# PulsePath: Two-Stage Generative Waveform Refinement for Robust Remote Photoplethysmography Estimation

**DOI:** 10.3390/s26196105

**Published:** 2026-09-26

**Authors:** Juha Park, Seungmin Oh, Sang Jun Lee

**Affiliations:** Division of Electronic Engineering, Jeonbuk National University, 567 Baekje-daero, Deokjin-gu, Jeonju 54896, Republic of Korea; juhapark@jbnu.ac.kr (J.P.); osm021102@naver.com (S.O.)

**Keywords:** remote photoplethysmography, waveform refinement, flow matching, residual diffusion, robust pulse estimation, cross-dataset generalization

## Abstract

Remote photoplethysmography (rPPG) estimates pulse signals from facial videos, but motion, illumination variation, exposure control, and compression can distort the waveform used for heart-rate (HR) estimation. The Plane-Orthogonal-to-Skin (POS) waveform provides an observed physiological source that often preserves recording-specific periodic structure. Existing methods commonly regress the complete waveform directly or reconstruct it from a stochastic state without using this source as the starting point. We propose PulsePath, a two-stage framework that transports POS toward paired-contact photoplethysmography (PPG) and models only the residual remaining after deterministic inference. Stage A performs conditional Flow Matching (FM) from POS using a six-channel multiscale spatiotemporal map and a 25-channel recording-level frequency representation. Stage B fixes the deterministic FM estimate and reconstructs its remaining error with variance-preserving residual diffusion and velocity prediction. In intra-dataset testing, PulsePath achieves mean absolute error (MAE), root mean square error (RMSE), and Pearson correlation values of 0.46/0.94/0.99 on PURE and 0.29/0.80/0.99 on UBFC-rPPG. In cross-dataset testing, PulsePath achieves an MAE/RMSE of 0.23/0.63 when trained on UBFC-rPPG and tested on PURE, and 1.13/2.49 when trained on PURE and tested on UBFC-rPPG. The additional analyses show protocol-dependent component effects and training-run variability; the reported HR results do not establish waveform fidelity.

## 1. Introduction

Remote photoplethysmography (rPPG) estimates cardiovascular activity, including heart rate (HR), from pulse-induced color changes in facial videos [1,2]. Because it requires no contact sensor, rPPG is useful for remote monitoring and camera-based human sensing. The physiological color variation is weak relative to head motion, illumination changes, exposure control, and compression artifacts. These disturbances can preserve a dominant HR frequency while corrupting amplitude, phase, and local temporal structure. This work studies waveform refinement toward paired-contact photoplethysmography (PPG) as a modeling strategy for recording-level HR estimation.

Classical color projection methods provide a structured starting point for this refinement problem. The Plane-Orthogonal-to-Skin (POS) method projects normalized red, green, and blue (RGB) traces onto a plane orthogonal to the skin-tone direction [2]. The resulting signal remains sensitive to motion, lighting, and camera noise. Nevertheless, POS often retains periodicity and coarse temporal organization specific to the observed recording. We select POS because it is training free, deterministic, and provides a waveform that can serve as an explicit source for FM. Regional color evidence and frequency context from the complete recording are used to compensate for information missing from this one-dimensional source.

Most learning-based rPPG methods map video representations directly to the complete target signal [3,4,5,6]. This formulation is effective for direct estimation, but it does not expose an interpretable source waveform inside the prediction path. Generative approaches model waveform distributions with adversarial learning, diffusion, or flow transport [7,8,9,10,11]. These methods may denoise a classical estimate or reconstruct the complete target waveform from a stochastic state. They do not combine deterministic transport from POS with stochastic modeling restricted to the residual that remains after transport. This distinction motivates a formulation that separates source transport from residual refinement.

The key insight is to decompose waveform refinement according to the physiological information already present in POS. Unlike an independent noise sample, POS retains periodic structure derived from the observed recording. This structure provides a meaningful initial state for learning paired transport toward contact PPG. The deterministic FM prediction can nevertheless retain fine-scale errors under motion, illumination variation, and compression. Rather than regenerating the complete waveform, the remaining error can be isolated and modeled as a residual distribution.

We propose PulsePath, a generative waveform refinement framework with two stages for robust rPPG estimation. Stage A replaces the simple noise source commonly used in generative Flow Matching (FM) with the observed POS waveform and learns paired transport toward contact PPG. Regional color evidence with six channels and frequency context from the complete recording condition the velocity field. Two Euler updates guided by the source condition produce the deterministic FM estimate ${\hat{y}}_{F}$. Stage B fixes this estimate and applies diffusion only to the residual between ${\hat{y}}_{F}$ and contact PPG. At inference, ten waveforms corrected by residual diffusion are evaluated together with the unmodified FM estimate using recording-level spectral consistency without target labels. PulsePath retains competitive intra-dataset HR accuracy and shows low cross-dataset error for UBFC-rPPG→PURE, while the reverse transfer direction remains competitive and exhibits greater variability in the additional multi-seed evaluation. The main contributions are summarized below.

- **FM transport from an observed POS waveform.** Unlike standard generative FM formulations that typically initialize transport from a simple noise distribution, PulsePath defines POS as the initial state of the transport path. This formulation learns a direct transformation from a video-derived physiological waveform to paired-contact PPG while using the temporal structure already contained in POS.
- **Residual diffusion conditioned on the FM estimate.** Instead of reconstructing the complete waveform with diffusion, PulsePath fixes the inference FM estimate and models only the remaining residual. This decomposition assigns the primary waveform transformation to FM and restricts stochastic generation to unresolved waveform error.

## 2. Related Work

### 2.1. Physiological Signal Estimation from Facial Videos

Classical rPPG methods recover pulse information from spatially averaged color traces. Green channel methods [1] provide a simple baseline by exploiting the strong visible light response of blood-volume variation, although they remain sensitive to motion and illumination. Independent component analysis (ICA) [12] separates periodic components across color channels and improves robustness when the mixed sources are sufficiently distinct. CHROM [13] suppresses motion-related chrominance variation, while PBV [14] uses a predefined pulse signature to improve motion robustness. POS [2] projects normalized RGB traces onto a plane orthogonal to the skin-tone direction and produces a deterministic pulse waveform without training. LGI [15] and OMIT [16] further reduce nuisance variation through invariant or orthogonal color transformations. We choose POS because it is efficient, interpretable, and provides an observed waveform whose periodic structure is suitable as the initial state of FM.

Learning-based methods replace fixed color transforms with trainable spatial and temporal representations. DeepPhys [3] introduced appearance-guided attention to improve the extraction of subtle physiological changes. TS-CAN [4] incorporated temporal shifts to reduce computation while preserving temporal modeling, and EfficientPhys [17] further simplified preprocessing for faster inference. PhysNet [18] used a spatiotemporal convolutional network to recover pulse waveforms directly from video. PhysFormer [5] combined temporal difference attention with frequency supervision to improve temporal and spectral modeling. Contrast-Phys [19] and SiNC [20] reduced dependence on aligned labels, while NEST [6] improved generalization across acquisition domains. Unlike these direct estimators, PulsePath uses the observed POS waveform as the explicit start of a generative transport path.

### 2.2. Conditioning for rPPG Waveform Refinement

Map-based representations summarize facial color changes over regions and time. RhythmNet [21] organized regional traces as a spatiotemporal map and improved HR estimation by preserving spatially distributed pulse information. Cross-verified feature disentangling [22] introduced a multiscale spatiotemporal map (MSTMap) to separate physiological features from motion and illumination interference. Dual-Path TokenLearner [23] modeled regional and temporal dependencies through separate token paths, improving the use of broader context. These representations retain spatial evidence that disappears after global color averaging. Accordingly, PulsePath uses an MSTMap with six channels as auxiliary conditioning while preserving POS as the primary waveform source.

Frequency information provides a complementary description of quasi-periodic cardiac activity. PhysFormer [5] and SiNC [20] use spectral constraints to stabilize physiological estimation and reduce temporally implausible predictions. FreqPhys [10] incorporates physiological filtering, spectrum modulation, and diffusion conditioned on frequency information to improve robustness across challenging conditions. A single dominant frequency cannot represent temporal variation or uncertainty across a complete recording. We therefore construct a frequency condition with 25 channels from the complete POS waveform using reconstructed subbands, a relative frequency vote distribution, and confidence information. This condition complements the local POS waveform with frequency context at the recording level.

### 2.3. Generative Waveform Modeling

Generative rPPG methods refine classical signals or reconstruct complete waveform distributions. PulseGAN [7] improves the realism of CHROM waveforms through adversarial, temporal, and spectral losses. Dual-GAN [8] jointly models blood-volume pulse (BVP) estimation and the noise distribution to improve feature disentanglement.

PhysDiff [9] uses conditional diffusion with dynamicity modeling and multiple hypotheses to improve robust rPPG reconstruction. FreqPhys [10] adds explicit frequency priors to diffusion and strengthens physiological consistency across domains. These approaches demonstrate the value of probabilistic waveform modeling, but their generators remain responsible for reconstructing the complete signal.

Flow Matching (FM) [24] learns a velocity field that varies with time along a prescribed probability path. Rectified Flow [25] favors straighter paths that require fewer numerical integration steps during generation. PhysFlow [11] applies separate rectified flow-fields to trend and amplitude components of rPPG for efficient waveform generation. Our FM path instead starts from the observed POS waveform and terminates at paired-contact PPG. The deterministic FM estimate is fixed before residual diffusion training. PulsePath therefore narrows the role of diffusion from complete signal reconstruction to correction of the residual left by FM.

## 3. Proposed Method

Figure 1 presents the architecture of PulsePath. During Stage A, the FM model transports the observed POS waveform toward paired-contact PPG. After Stage A training, the FM model is fixed and produces the deterministic estimate ${\hat{y}}_{F}$ for residual learning. Stage B uses a separate residual velocity predictor whose feature layers are initialized from the trained FM velocity predictor. The two losses are optimized sequentially and are never backpropagated at the same time. At inference, selection without target labels compares ${\hat{y}}_{F}$ with ten waveforms corrected by residual diffusion.

Throughout the manuscript, s denotes the POS source, ${\hat{y}}_{F}$, the deterministic estimate, and $r_{0}=y-{\hat{y}}_{F}$, the training residual. The notation $Y^{\left(h\right)}$ denotes a residual-corrected candidate, whereas $\hat{y}=Y^{\left(h^{★}\right)}$ denotes the final selected output; $Y^{\left(0\right)}={\hat{y}}_{F}$ is also eligible for selection.

### 3.1. Shared Physiological Representation

Each recording is divided into N∈{4,5,6,7,8} temporally ordered chunks, where *N* denotes the number of chunks. Each chunk contains T=300 samples corresponding to 10 s at 30 frames per second. The POS source and paired-contact PPG are denoted by $s,y\in R^{N\times T}$. The MSTMap is denoted by $M\in R^{N\times 6\times R\times T}$, where R=63 is the number of facial regions. Its six channels comprise RGB, luminance *Y*, and chrominance *U* and *V*, where *Y* represents luminance and *U* and *V* represent chrominance. Each regional trace is linearly scaled to [0,255] over time in the cached input.

The POS waveform is extracted over the complete recording using a window of 48 frames with a hop of one frame at 30 Hz. Each window is centered by its mean and overlap added after the POS projection [2]. The resulting waveform is processed by smoothness prior detrending with λ=100, where λ is the detrending regularization parameter. A second-order Butterworth bandpass over 0.6–3.3 Hz is then applied with zero-phase filtering. The complete recording is standardized to zero mean and unit population variance before chunking. No additional filtering or normalization is applied to individual chunks.

The conditions $C_{F},C_{D}\in R^{N\times 32\times R\times T}$ combine the MSTMap, a waveform repeated across regions, and a frequency representation with 25 channels along the channel dimension. The repeated waveform is s in Stage A and ${\hat{y}}_{F}$ in Stage B. The frequency representation is constructed from the complete POS recording in Stage A and from the complete FM estimate in Stage B. It contains eight reconstructed subband signals, sixteen discrete cosine coefficients of a relative frequency vote distribution with 65 bins, and one confidence channel. The candidate scale spans [0.8,1.25] around the dominant frequency of the waveform used in the corresponding stage. The reference waveform y is not included in either condition.

Before velocity prediction, the current waveform state and the corresponding condition are joined along the channel dimension and projected to feature tokens. Let $V_{\theta_{F}}$ and $V_{\theta_{D}}$ denote the FM and residual velocity predictors, respectively. Both predictors use the spatial temporal hybrid velocity block adapted from PhysDiff [9] to model interactions across facial regions and time. Region, temporal position, and sinusoidal time embeddings are added to the projected tokens. A single condition drop decision is sampled per recording with probability $p_{\mathrm{drop}}=0.10$ and is shared by all chunks. When the condition is dropped, the MSTMap and frequency channels are set to zero while the repeated waveform is retained.

In the standard training configuration, one recording-level dropout decision jointly removes the MSTMap and all 25 frequency channels with probability 0.10. The two sources are not independently dropped in that configuration; the conditioning ablations instead remove the selected pathways throughout training. The waveform channel is retained, so the source-conditioned branch is not fully unconditional.

### 3.2. Stage A: FM Transport from POS

For chunk *i*, we sample $z_{i}∼N\left(0,1\right)$ and define $\tau_{i}=\mathrm{sigmoid}\left(z_{i}\right)\in \left(0,1\right)$ as the FM time. The variable $x_{\tau_{i},i}\in R^{T}$ denotes the waveform state at time $\tau_{i}$ along the straight path from POS to contact PPG.

(1) $$x_{\tau_{i},i}=s_{i}+\tau_{i}\left(y_{i}-s_{i}\right).$$

At $\tau_{i}=0$, the path starts from the observed POS waveform rather than an independent noise sample. The target velocity is $v_{F,i}=y_{i}-s_{i}$, and the predicted velocity is ${\hat{v}}_{F,i}=V_{\theta_{F}}\left(x_{\tau_{i},i},C_{F,i},\tau_{i}\right)$. The FM loss is defined as follows:

(2) $$L_{\mathrm{FM}}=\frac{1}{N}\sum_{i=1}^{N}\left(1+\tau_{i}^{2}\right)\frac{1}{T}{∥{\hat{v}}_{F,i}-v_{F,i}∥}_{2}^{2}.$$

The factor $1+\tau_{i}^{2}$ gives greater weight to errors near contact PPG. The local reconstruction ${\tilde{y}}_{F}=s+{\hat{v}}_{F}$ is used for waveform supervision at the chunk level.

Condition dropout trains a full-condition branch and a source-condition branch that retains POS while setting the MSTMap and frequency channels to zero. At inference, condition dropout is disabled and both branches are evaluated explicitly using guidance inspired by classifier-free guidance [26]. Let ${\hat{v}}_{F}^{\mathrm{full}}\left(x,\tau \right)$ and ${\hat{v}}_{F}^{\mathrm{src}}\left(x,\tau \right)$ denote predictions from the full and source conditions, respectively. The guidance weight is denoted by $w_{\mathrm{CFG}}$ and is set to 1.5. The guided velocity is defined as follows:

(3) $${\tilde{v}}_{F}\left(x,\tau \right)={\hat{v}}_{F}^{\mathrm{src}}\left(x,\tau \right)+w_{\mathrm{CFG}}\left({\hat{v}}_{F}^{\mathrm{full}}\left(x,\tau \right)-{\hat{v}}_{F}^{\mathrm{src}}\left(x,\tau \right)\right).$$

Starting from $x^{\left(0\right)}=s$, the first Euler update evaluates the guided velocity at τ=0.

(4) $$x^{\left(1\right)}=x^{\left(0\right)}+\frac{1}{2}{\tilde{v}}_{F}\left(x^{\left(0\right)},0\right).$$

The second update evaluates it at τ=1/2.

(5) $$x^{\left(2\right)}=x^{\left(1\right)}+\frac{1}{2}{\tilde{v}}_{F}\left(x^{\left(1\right)},1/2\right).$$

The inference FM estimate is ${\hat{y}}_{F}=x^{\left(2\right)}$. Each Euler step evaluates the velocity predictor once with each condition, resulting in four network evaluations in total.

The same guidance weight $w_{\mathrm{CFG}}=1.5$ is used in both Euler updates, with conditioning dropout disabled during inference.

Our formulation follows the Flow Matching principle of learning a time-dependent velocity field along a prescribed path, but uses paired POS and contact-PPG observations as the transport endpoints rather than an independent noise source. Unlike direct residual regression, which predicts the endpoint correction through a single mapping, this formulation models intermediate states along the POS-to-PPG path and integrates the velocity field from the observed POS source at inference. We use this distinction as a structural motivation rather than as evidence of universal superiority over direct residual regression.

### 3.3. Physiological Supervision Across Stages

Both stages use the same clean waveform loss. The loss combines Pearson correlation, power spectral density (PSD), soft HR, and dynamicity terms.

(6) $$L_{\mathrm{clean}}=L_{\mathrm{Pearson}}+0.10L_{\mathrm{PSD}}+0.05L_{\mathrm{HR}}+0.10L_{\mathrm{dyn}}.$$

Let $\rho_{w}\left(\cdot ,\cdot \right)$ denote waveform Pearson correlation; the Pearson term is $N^{-1}\sum_{i}\left[1-\rho_{w}\left({\hat{y}}_{i},y_{i}\right)\right]$. For the PSD term, each chunk is centered, multiplied by a Hann window, transformed with a fast Fourier transform using 2048 points, restricted to 0.6–3.3 Hz, and normalized to sum to one. Let $D_{\mathrm{KL}}$ denote the Kullback–Leibler divergence; with $m_{i}=\left(p_{{\hat{y}}_{i}}+p_{y_{i}}\right)/2$, the PSD loss averages $\left[D_{\mathrm{KL}}\left(p_{{\hat{y}}_{i}}∥m_{i}\right)+D_{\mathrm{KL}}\left(p_{y_{i}}∥m_{i}\right)\right]/\left(2\log2\right)$ over chunks. For chunk *i*, the soft HR weights are $w_{ij}={\mathrm{softmax}}_{j}\left(\log p_{ij}/\tau_{\mathrm{HR}}\right)$ with $\tau_{\mathrm{HR}}=0.25$, and the differentiable estimate is $60\sum_{j}w_{ij}f_{j}$, where $f_{j}$ is the frequency in hertz at bin *j*. The HR loss applies Smooth L1 after dividing the predicted and reference HR values by 10, while Stage A and Stage B apply $L_{\mathrm{clean}}$ to ${\tilde{y}}_{F}$ and ${\hat{y}}_{D}$, respectively.

For a waveform $z=(z_{1},\ldots ,z_{T})$, let $\Delta z_{i}=z_{i+1}-z_{i}$ for i=1,…,T−1. The final difference is repeated as $\Delta z_{T}=\Delta z_{T-1}$ to preserve temporal alignment. The amplitude variation descriptor is defined as follows:

(7) $$a_{i}\left(z\right)=\sqrt{{\left(\Delta z_{i}\right)}^{2}+1}.$$

The signed trend descriptor is defined as follows:

(8) $$d_{i}\left(z\right)=\frac{\Delta z_{i}}{\sqrt{{\left(\Delta z_{i}\right)}^{2}+1}}.$$

The dynamicity loss is defined as follows:

(9) $$L_{\mathrm{dyn}}\left(\hat{y},y\right)=\mathrm{MSE}\;\left(d\left(\hat{y}\right),d\left(y\right)\right)+\mathrm{MSE}\;\left(a\left(\hat{y}\right),a\left(y\right)\right).$$

The mean squared errors are averaged over chunks and temporal samples.

The recording-level ranking loss is computed after joining the *N* predicted chunks in temporal order. Let $p_{j}$ denote the normalized predicted PSD probability at frequency bin *j*, and let $j^{★}$ denote the dominant bin of the detached reference PSD. Float32 machine epsilon is added before probability normalization and is applied again as a lower bound before each logarithm. The soft target assigns 0.8 to $j^{★}$ and 0.1 to each adjacent index after clamping the indices to the valid range. The cross-entropy (CE) loss is defined as follows:

(10) $$L_{\mathrm{CE}}=-\sum_{j}q_{j}\log p_{j}.$$

Let $p_{\mathrm{comp}}=\max_{j\neq j^{★}}p_{j}$, and let $P_{y}^{\left(1\right)}$ and $P_{y}^{\left(2\right)}$ be the largest and second largest detached reference spectral powers. Using the indicator function 1[·], the ambiguity aware margin loss is

(11) $$L_{\mathrm{margin}}=1\;\left[\frac{P_{y}^{\left(1\right)}}{P_{y}^{\left(2\right)}}\geq 1.05\right]\mathrm{softplus}\;\left(\log p_{\mathrm{comp}}-\log p_{j^{★}}+m\right).$$

The complete ranking loss is $L_{\mathrm{rank}}=L_{\mathrm{CE}}+0.5L_{\mathrm{margin}}$.

Stage A uses $m_{A}=\log1.25$. The Stage B margin at epoch e∈{1,…,30} is

(12) $$m_{B}\left(e\right)=\log1.25+\frac{e-1}{29}\left(\log1.5-\log1.25\right).$$

The Stage A loss is

(13) $$L_{A}=L_{\mathrm{FM}}+L_{\mathrm{clean}}\left({\tilde{y}}_{F},y\right)+0.07L_{\mathrm{rank}}\left({\hat{y}}_{F},y;m_{A}\right).$$

The clean loss uses the local reconstruction, whereas the ranking loss uses the inference FM estimate. The Stage B loss uses the same ranking definition with $m_{B}\left(e\right)$.

### 3.4. Stage B: Residual Diffusion Conditioned on the FM Estimate

After Stage A, the trained feature layers of the FM velocity predictor initialize a separate residual velocity predictor. The FM and residual predictors do not share parameters, and the FM output head is not copied to the residual predictor. The original FM predictor remains fixed throughout Stage B. The FM estimate ${\hat{y}}_{F}$ is recomputed using Equations (4) and (5) and is detached from the computation graph. The clean residual is $r_{0}=y-{\hat{y}}_{F}$, with no gradient propagated through ${\hat{y}}_{F}$. The residual predictor is optimized only for this remaining error.

One time t∼U[0.02,0.98) is sampled per recording and shared across its chunks. With $\alpha_{t}=\cos\left(\pi t/2\right)$ and $\sigma_{t}=\sin\left(\pi t/2\right)$, the variance-preserving (VP) process is [27,28]

(14) $$r_{t,i}=\alpha_{t}r_{0,i}+\sigma_{t}\epsilon_{i}.$$

Here, $\epsilon_{i}∼N\left(0,I\right)$, where I is the identity covariance matrix. Using velocity parameterization [29], the target is $v_{D,i}=\alpha_{t}\epsilon_{i}-\sigma_{t}r_{0,i}$. The predicted velocity is ${\hat{v}}_{D,i}=V_{\theta_{D}}\left(r_{t,i},C_{D,i},t\right)$. The clean residual estimate is ${\hat{r}}_{0,i}=\alpha_{t}r_{t,i}-\sigma_{t}{\hat{v}}_{D,i}$. The corrected waveform is ${\hat{y}}_{D,i}={\hat{y}}_{F,i}+{\hat{r}}_{0,i}$.

Minimum signal-to-noise ratio (Min-SNR) weighting uses $w_{\gamma }\left(t\right)=\min\left(\alpha_{t}^{2},\gamma \sigma_{t}^{2}\right)$ with γ=5 [30]. The residual velocity loss is defined as follows:

(15) $$L_{v}=\frac{1}{N}\sum_{i=1}^{N}w_{\gamma }\left(t\right)\frac{1}{T}{∥{\hat{v}}_{D,i}-v_{D,i}∥}_{2}^{2}.$$

The complete Stage B loss at epoch *e* is defined as follows:

(16) $$L_{B}\left(e\right)=L_{v}+L_{\mathrm{clean}}\left({\hat{y}}_{D},y\right)+0.07L_{\mathrm{rank}}\left({\hat{y}}_{D},y;m_{B}\left(e\right)\right).$$

Only the residual predictor parameters $\theta_{D}$ are updated by $L_{B}\left(e\right)$. No direct noise prediction or residual reconstruction mean squared error is added.

### 3.5. Inference Without Target Labels

Inference first computes the deterministic FM estimate ${\hat{y}}_{F}$. Stage B generates H=10 residual hypotheses from independent Gaussian initializations. Each hypothesis uses deterministic Denoising Diffusion Implicit Model (DDIM) sampling with η=0 under the fixed condition $C_{D}$ [31]. The time sequence is $t_{k}=1-k/10$ for k=0,…,10, and the residual predictor is evaluated at $t_{0},\ldots ,t_{9}$. The final update reaches $t_{10}=0$ without another network evaluation or clipping at the time boundaries. The final residual produces $Y^{\left(h\right)}={\hat{y}}_{F}+{\hat{r}}_{0}^{\left(h\right)}$, and $Y^{\left(0\right)}={\hat{y}}_{F}$ retains the unmodified FM estimate as an additional candidate.

Each candidate is centered by its mean and divided by its population standard deviation over the complete recording, with a floor of $10^{-6}$. The standardized waveform is divided into J=3 segments of equal length, and trailing samples are discarded when necessary. Each segment is centered again and transformed with an unwindowed real fast Fourier transform whose length equals the segment length. The selector uses the unnormalized power ${\left|\mathrm{rFFT}\right|}^{2}$ after removing only the direct current bin. Let $P_{h,j}$ denote the segment power spectrum for candidate *h* and segment *j*. The candidate score is defined as follows:

(17) $$S_{h}=1-\sum_{j=1}^{J-1}{∥P_{h,j+1}-P_{h,j}∥}_{2}.$$

The selected index is $h^{★}={\arg\;\max}_{h\in \{0,\ldots ,H\}}S_{h}$, and the final waveform is $\hat{y}=Y^{\left(h^{★}\right)}$ without using contact PPG or an HR label.

Because frequency conditioning and final candidate selection use information from the complete recording, the current implementation operates as an offline recording-level method rather than a causal streaming estimator.

## 4. Experimental Results

### 4.1. Datasets and Subject Splits

We evaluate PulsePath on the public PURE and UBFC-rPPG datasets [32,33]. The evaluation uses subject-disjoint training, validation, and test sets. Intra-dataset testing uses separate subjects from the same dataset. Cross-dataset testing trains and validates on the source dataset and evaluates only on the target dataset. Table 1 reports the subjects used in each protocol.

### 4.2. Training and Evaluation Details

Stage A and Stage B are each trained for 30 epochs using separate AdamW optimizers with learning rates of $10^{-4}$ and $5\times 10^{-5}$, respectively. Weight decay is set to zero, and the gradient norm is clipped to 1.0. Each optimization step uses one complete recording, with N∈{4,5,6,7,8} consecutive chunks of 300 samples forming the tensor batch dimension. All experiments are conducted on a single NVIDIA GeForce RTX 4090 GPU. Table 2 reports the original seed 102 intra-dataset results. Table 3 reports the seed 102 cross-dataset evaluation using the fixed checkpoints (Stage A epoch 1 and Stage B epoch 3), while the additional three-seed frozen-checkpoint evaluation is summarized as mean ± sample standard deviation in Table 4. No target-test information was used for checkpoint or model selection in either evaluation. For evaluation, all chunks from a recording are joined to obtain one HR estimate. Performance is measured using mean absolute error (MAE), root mean square error (RMSE), and Pearson correlation $\rho_{\mathrm{HR}}$, with MAE and RMSE reported in beats per minute.

For recording-level HR evaluation, the predicted and reference waveforms undergo the same smoothness-prior detrending (λ=100) and zero-phase band-pass filtering over 0.6–3.3 Hz. A periodogram is computed over the complete recording using a boxcar (rectangular) window and the next power-of-two FFT length, $N_{\mathrm{FFT}}$. The frequency-bin spacing is $f_{s}/N_{\mathrm{FFT}}$ Hz. HR is calculated as $60f_{\mathrm{peak}}$ bpm, where $f_{\mathrm{peak}}$ is the frequency of the maximum-power bin within 0.6–3.3 Hz. The same procedure is applied to the predicted and reference waveforms.

The baseline values in Table 2 and Table 3 are taken from the cited publications rather than reproduced under a common evaluation pipeline. PulsePath follows the subject-disjoint protocols in Table 1, and differences in evaluation settings should therefore be considered when interpreting these comparisons.

PulsePath contains 386,190 parameters. On an NVIDIA RTX 4090 in FP32 with one recording per inference batch, processing a 60 s recording took approximately 0.14 s for Stage A and 7.02 s for the complete configuration with ten residual hypotheses and ten DDIM steps. The complete configuration required approximately $6.95\times 10^{12}$ profiler-estimated FLOPs. Timing was measured under concurrent GPU workload and excludes video decoding and preprocessing.

The computational profile used PyTorch 2.1.2 with CUDA 12.1, 50 warm-up runs, and 200 GPU-synchronized timed runs. For the full 60 s configuration, the mean latency was 7.024±0.160 s, the model-only processing throughput was 256.3 frames/s, and peak GPU memory was 357.7 MiB. The checkpoint occupied 1,618,174 bytes. These are offline processing measurements, not streaming throughput or end-to-end application latency.

For the additional frozen-checkpoint evaluation, checkpoints were selected using source-validation MAE +0.25× RMSE, with the earliest epoch used to break ties. Checkpoints were frozen before evaluation on the fixed test splits in Table 1; no target-domain fine-tuning or reference-guided candidate selection was performed. The base inference settings (H=10, ten DDIM steps, and $w_{\mathrm{CFG}}=1.5$) were held fixed. Subsequent sensitivity checks were diagnostic and were not used to replace the reported base settings.

### 4.3. Intra-Dataset Testing

Table 2 reports intra-dataset HR estimation on PURE and UBFC-rPPG. On PURE, PulsePath achieves an MAE of 0.46, an RMSE of 0.94, and $\rho_{\mathrm{HR}}=0.99$. These are the lowest MAE and RMSE values among the methods listed in the table. The improvements over Contrast-Phys+ are modest, with reductions of 0.02 in MAE and 0.04 in RMSE. The value of $\rho_{\mathrm{HR}}$ matches the highest-reported value shared by several methods. These results indicate that the proposed waveform-refinement formulation maintains strong intra-dataset HR accuracy on PURE.

On UBFC-rPPG, PulsePath achieves an MAE of 0.29, an RMSE of 0.80, and $\rho_{\mathrm{HR}}=0.99$. Its MAE is the second lowest among the listed methods, following Contrast-Phys+ at 0.21. Its RMSE is comparable to Contrast-Phys+ but remains above the values reported by Li and Yin and PhysFormer. The value of $\rho_{\mathrm{HR}}$ matches the highest-reported value in the table. Overall, PulsePath provides competitive intra-dataset performance on UBFC-rPPG while preserving an explicit waveform-refinement path from POS to the final estimate.

### 4.4. Cross-Dataset Testing

Table 3 reports the seed 102 cross-dataset comparison using the fixed checkpoints. For UBFC-rPPG→PURE, PulsePath achieves an MAE of 0.23 and an RMSE of 0.63, which are the lowest-listed errors among the compared methods, although differences in evaluation protocols should be considered when interpreting the comparison. For PURE→UBFC-rPPG, PulsePath achieves an MAE of 1.13 and an RMSE of 2.49, remaining competitive with the listed methods. The additional source-validation-selected three-seed evaluation in Table 4 characterizes training-run variability separately and shows larger variability for PURE→UBFC-rPPG.

### 4.5. Additional Analyses

Table 4 reports the final selected PulsePath output across three training seeds for two intra-dataset and two cross-dataset protocols. Training-seed variability is larger for PURE→UBFC-rPPG than for the intra-dataset settings.

Table 5 summarizes matched conditioning ablations under the intra-dataset protocols. Check marks indicate the conditioning pathways retained in each configuration. Removing both auxiliary pathways increased HR MAE in both datasets. Removing direct MSTMap conditioning showed dataset-dependent effects, whereas removing frequency conditioning left the aggregate HR MAE unchanged.

Table 6 reports the final output under the PURE intra-dataset protocol. The mean HR MAE across three training seeds was below 0.9 bpm for each evaluated acquisition condition, showing low HR error within this test setting.

Training-seed standard deviations quantify run-to-run variability on fixed test subjects and are not subject-level confidence intervals. For the additional frozen-checkpoint evaluation, 95% confidence intervals for final-output HR MAE and RMSE were computed separately for each seed using 10,000 subject-cluster bootstrap resamples, retaining all recordings from each sampled subject. The intervals are reported in Table A1 (Appendix A). Formal paired significance against the literature baselines is not claimed because their subject-level predictions were not evaluated under the same pipeline.

We also compared Stage A, individual residual-corrected outputs, their sample-wise mean waveform, and the proposed selected output. No evaluated strategy consistently achieved the lowest HR MAE across all protocols. The corresponding numerical results are provided in Table A2 in Appendix B. The comparison between the deterministic estimate and the final output assesses residual generation and selection jointly, rather than isolating either operation. The conditioning ablations are limited to the intra-dataset protocols.

Additional sensitivity checks varied H∈{1,3,5,10} across three training seeds and the DDIM step count K∈{2,5,10} at a fixed seed-100 checkpoint. HR changes were protocol-dependent and did not establish one universally preferable setting. In the separate 60 s computational profile, reducing the configuration to H=1, K=10 or H=10, K=2 reduced model-only inference time to approximately 0.83 s or 1.56 s, respectively, compared with 7.02 s for H=10, K=10.

Exploratory seed-100 checks used $w_{\mathrm{CFG}}\in \left\{0,1,1.5,2\right\}$. Final HR MAE was unchanged to the reported precision in the intra-dataset settings but varied in the cross-dataset settings. Alternative weights were evaluated in separate fixed-noise runs, while 1.5 used the base candidate pool. These checks therefore do not isolate the weight from every sampling difference and do not establish 1.5 as an optimal value.

## 5. Discussion

The current evaluation measures recording-level HR accuracy and does not establish accurate recovery of detailed PPG morphology or individual beat timing. The additional conditioning ablations show protocol-dependent component effects. The condition-specific PURE analysis is limited to the acquisition conditions represented in this dataset, and the experiments do not cover the full range of cameras, lighting conditions, skin tones, and motion patterns encountered in practice. Generating ten residual hypotheses with ten DDIM steps also introduces additional inference cost. Small differences in a single run should not be interpreted as consistent superiority across training seeds. The spectral-consistency selector may favor stable periodic signals, and preservation of within-recording HR variability remains to be evaluated. These limitations define the scope of the evidence provided by the present experiments.

Potential failure modes include severe motion, poor illumination, face-tracking errors, and low-quality or compressed video. These are anticipated limitations rather than experimentally verified failure categories. Participant-diversity and skin-tone-specific performance were not separately evaluated, and no additional dataset beyond PURE and UBFC-rPPG is reported in this study.

## 6. Conclusions

We presented PulsePath, a generative waveform refinement framework with two stages for robust rPPG estimation. Stage A performs FM transport from an observed POS waveform toward contact PPG, and Stage B models only the residual around the fixed FM estimate. Selection without target labels chooses among the FM estimate and ten waveforms corrected by residual diffusion. In intra-dataset testing, PulsePath achieves an MAE of 0.46, an RMSE of 0.94, and $\rho_{\mathrm{HR}}=0.99$ on PURE, and an MAE of 0.29, an RMSE of 0.80, and $\rho_{\mathrm{HR}}=0.99$ on UBFC-rPPG. For UBFC-rPPG→PURE, it achieves an MAE of 0.23 and an RMSE of 0.63. For PURE→UBFC-rPPG, it achieves an MAE of 1.13 and an RMSE of 2.49. These results show competitive recording-level HR performance under the reported protocols, while the additional analyses indicate protocol-dependent component effects and training-run variability. Future work will examine more efficient residual sampling, broader acquisition conditions, and additional measures of waveform quality.

## Figures and Tables

**Figure 1:**
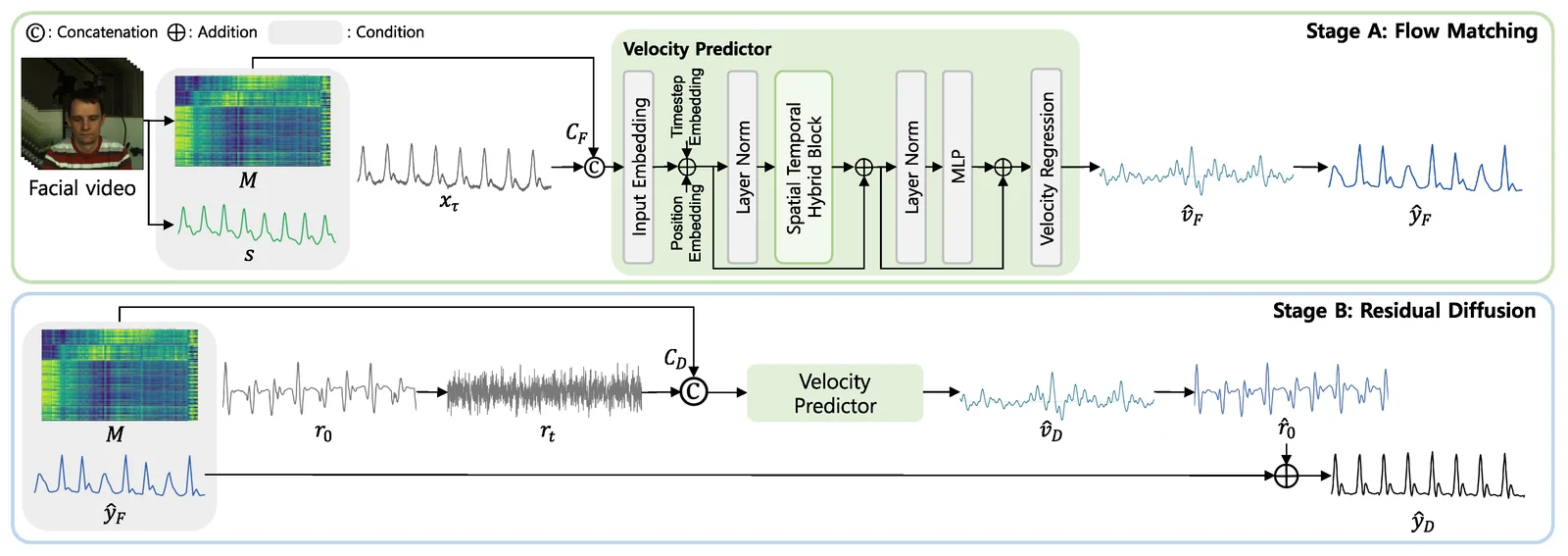
Overview of PulsePath. Stage A transports the observed POS waveform toward paired-contact PPG using conditional Flow Matching, with two Euler updates used for numerical integration. Stage B models the remaining residual around the fixed FM estimate using residual diffusion. At inference, the final waveform is selected from the FM estimate and ten residual-corrected candidates without reference PPG.

**Table 1:** Subject-disjoint splits used for intra-dataset and cross-dataset testing.

Protocol	Training Subjects	Validation Subjects	Test Subjects
PURE	PURE: 1, 2, 3, 4	PURE: 5, 6	PURE: 7, 8, 9, 10
UBFC-rPPG	UBFC-rPPG: 1, 10, 11, 12, 13, 14, 15, 16, 17, 18, 20, 22, 23, 24, 25, 26, 27, 3, 30, 31, 32, 33, 34, 35	UBFC-rPPG: 36, 37, 38, 39, 4, 40	UBFC-rPPG: 41, 42, 43, 44, 45, 46, 47, 48, 49, 5, 8, 9
PURE→UBFC-rPPG	PURE: 1–8	PURE: 9, 10	All 42 UBFC-rPPG subjects
UBFC-rPPG→PURE	UBFC-rPPG: 1, 10, 11, 12, 13, 14, 15, 16, 17, 18, 20, 22, 23, 24, 25, 26, 27, 3, 30, 31, 32, 33, 34, 35, 36, 37, 38, 39, 4, 40, 41, 42, 43	UBFC-rPPG: 44, 45, 46, 47, 48, 49, 5, 8, 9	PURE: 1–10

**Table 2:** Intra-dataset testing results for heart-rate (HR) estimation on PURE and UBFC-rPPG. Mean absolute error (MAE) and root mean square error (RMSE) are reported in beats per minute, and $\rho_{\mathrm{HR}}$ denotes the Pearson correlation between estimated and reference HR values. The best and second-best results in each column are shown in bold and underline, respectively. Baseline values are taken from the cited publications, whereas PulsePath is evaluated using the protocols in Table 1.

Method	PURE			UBFC-rPPG		
	MAE↓	RMSE↓	$\rho_{\mathrm{HR}}$↑	MAE↓	RMSE↓	$\rho_{\mathrm{HR}}↑$
DeepPhys [3]	0.83	1.54	**0.99**	6.27	10.82	0.65
TS-CAN [4]	2.48	9.01	0.92	1.70	2.72	**0.99**
EfficientPhys [17]	–	–	–	1.14	1.81	**0.99**
PhysNet [18]	2.10	2.60	**0.99**	2.95	3.67	0.97
PhysFormer [5]	1.10	1.75	**0.99**	0.50	0.71	**0.99**
Li and Yin [34]	0.64	1.16	**0.99**	0.48	**0.64**	**0.99**
SiNC [20]	0.61	1.84	**0.99**	0.59	1.83	**0.99**
MaskFusionNet [35]	1.11	1.39	**0.99**	–	–	–
FactorizePhys [36]	3.08	13.84	0.91	2.08	3.63	0.98
Contrast-Phys+ [37]	0.48	0.98	**0.99**	**0.21**	0.80	**0.99**
PulsePath	**0.46**	**0.94**	**0.99**	0.29	0.80	**0.99**

**Table 3:** Cross-dataset testing results for HR estimation between UBFC-rPPG and PURE. The dataset before the arrow is used for training and validation, and the dataset after the arrow is used for testing. MAE and RMSE are reported in beats per minute, and $\rho_{\mathrm{HR}}$ denotes the Pearson correlation between estimated and reference HR values. The best and second-best results in each column are shown in bold and underline, respectively. Baseline values are taken from the cited publications, whereas PulsePath is evaluated using the protocols in Table 1. The PulsePath row reports the seed 102 fixed-checkpoint evaluation; the additional validation-selected multi-seed results are reported separately in Table 4.

Method	UBFC-rPPG→PURE			PURE→UBFC-rPPG		
	MAE↓	RMSE↓	*ρ*_HR_↑	MAE↓	RMSE↓	*ρ*_HR_↑
Green [1]	10.09	23.85	0.34	19.73	31.00	0.37
ICA [12]	4.77	16.07	0.72	16.00	25.65	0.44
CHROM [13]	5.77	14.93	0.81	4.06	8.83	0.89
LGI [15]	4.61	15.38	0.77	15.80	28.55	0.36
PBV [14]	3.92	12.99	0.84	15.90	26.40	0.48
POS [2]	3.67	11.82	0.88	4.08	7.72	0.92
OMIT [16]	4.69	15.82	0.76	15.96	28.57	0.36
DeepPhys [3]	5.54	18.51	0.66	1.21	2.90	**0.99**
TS-CAN [4]	3.69	13.80	0.82	1.30	2.87	**0.99**
EfficientPhys [17]	5.47	17.04	0.71	2.07	6.32	0.94
PhysNet [18]	8.06	19.71	0.61	0.98	2.48	**0.99**
PhysFormer [5]	12.92	24.36	0.47	1.44	3.77	0.98
Spiking-PhysFormer [38]	3.83	–	0.83	2.80	–	0.95
RhythmMamba [39]	1.98	6.51	0.96	0.95	1.83	**0.99**
StreamPPG [40]	2.00	7.93	0.94	**0.33**	**0.50**	**0.99**
PulsePath	**0.23**	**0.63**	**0.99**	1.13	2.49	**0.99**

**Table 4:** Recording-level HR performance of the final selected PulsePath output in the additional three-seed evaluation. Only the final selected output is reported. Intra-dataset and cross-dataset protocols are grouped separately. Values are mean ± sample standard deviation across training seeds 100, 101, and 102.

Evaluation	Protocol	MAE (bpm) ↓	RMSE (bpm) ↓
Intra-dataset	PURE	0.507±0.021	0.992±0.041
	UBFC-rPPG	0.415±0.112	1.026±0.249
Cross-dataset	PURE→UBFC-rPPG	2.923±2.766	5.320±4.295
	UBFC-rPPG→PURE	0.348±0.080	1.037±0.275

**Table 5:** Conditioning ablation under the intra-dataset protocols. A check mark (✓) indicates that the corresponding conditioning pathway is retained. Rows are ordered as no auxiliary conditioning, MSTMap only, frequency condition only, and both pathways. HR MAE is reported as mean ± sample standard deviation across three training seeds for PURE and two completed training seeds for UBFC-rPPG.

Direct MSTMap	Frequency Condition	PURE MAE (bpm) ↓	UBFC-rPPG MAE (bpm) ↓
		0.952±0.468	0.550±0.052
✓		0.507±0.021	0.476±0.052
	✓	0.525±0.053	0.403±0.156
✓	✓	0.507±0.021	0.476±0.052

**Table 6:** Condition-specific recording-level HR MAE of the final PulsePath output under the PURE intra-dataset protocol. Values are mean ± sample standard deviation across three training seeds.

Acquisition Condition	HR MAE (bpm) ↓
Steady	0.879±0.220
Talking	0.220±0.000
Slow Translation	0.146±0.254
Fast Translation	0.769±0.000
Small Rotation	0.366±0.127
Medium Rotation	0.659±0.381

## Data Availability

Publicly available datasets were analyzed in this study. The PURE dataset is available upon request from the official Technische Universität Ilmenau website: https://www.tu-ilmenau.de/en/university/departments/department-of-computer-science-and-automation/profile/institutes-and-groups/institute-of-computer-and-systems-engineering/group-for-neuroinformatics-and-cognitive-robotics/data-sets-code/pulse-rate-detection-dataset-pure, accessed on 4 August 2026. The UBFC-rPPG dataset is available from the official UBFC-rPPG website: https://sites.google.com/view/ybenezeth/ubfcrppg, accessed on 4 August 2026. The source code for this study will be made publicly available after publication.

## Appendix A. Subject-Level Uncertainty

Table A1 reports the subject-level uncertainty of the additional final-output evaluation. Intervals are computed independently for each training seed by resampling subjects with replacement and retaining all of their recordings. The test sets contain 4, 12, 42, and 10 subjects for PURE intra-dataset, UBFC-rPPG intra-dataset, PURE→UBFC-rPPG, and UBFC-rPPG→PURE, respectively. These intervals describe uncertainty over test subjects, not training-seed variability, and are not confidence intervals for the original single-seed literature-comparison entries in Table 2 and Table 3.

**Table A1:** 95% subject-cluster bootstrap confidence intervals for HR MAE and RMSE (bpm) of the final selected output in the additional evaluation, using 10,000 resamples per training seed. Only final-output metrics are shown.

Protocol	Metric	Seed 100	Seed 101	Seed 102
PURE (intra)	MAE	[0.220,0.769]	[0.146,0.970]	[0.256,0.641]
	RMSE	[0.567,1.303]	[0.359,1.572]	[0.595,1.213]
UBFC-rPPG (intra)	MAE	[0.073,1.025]	[0.000,1.172]	[0.000,0.732]
	RMSE	[0.254,1.501]	[0.000,2.226]	[0.000,1.343]
PURE→UBFC-rPPG	MAE	[3.809,8.747]	[0.732,2.386]	[0.502,1.904]
	RMSE	[6.351,13.669]	[1.864,4.180]	[1.479,3.466]
UBFC-rPPG→PURE	MAE	[0.167,0.732]	[0.091,0.559]	[0.119,0.483]
	RMSE	[0.616,1.820]	[0.396,1.533]	[0.380,1.070]

## Appendix B. Output-Selection Comparison

Table A2 reports the numerical output-selection comparison. The same frozen candidate set is used for Stage A, the ten residual-corrected candidates, their sample-wise mean waveform, and the final selected output. Values are recording-level HR MAE reported as mean ± sample standard deviation across training seeds 100, 101, and 102.

**Table A2:** Recording-level HR MAE (bpm) for Stage A, ten residual-corrected candidates, their sample-wise mean waveform, and the final selected output across the four evaluation protocols. Values are mean ± sample standard deviation across training seeds 100, 101, and 102. The same frozen candidate set is used for all strategy comparisons.

Strategy	PURE Intra	UBFC-rPPG Intra	PURE→UBFC-rPPG	UBFC-rPPG→PURE
Stage A (${\hat{y}}_{F}$)	0.421±0.000	0.439±0.000	1.172±0.151	0.628±0.552
Candidate $Y^{\left(1\right)}$	0.531±0.160	0.195±0.169	3.836±4.147	0.387±0.066
Candidate $Y^{\left(2\right)}$	0.421±0.000	0.366±0.220	3.327±3.298	0.345±0.106
Candidate $Y^{\left(3\right)}$	0.543±0.085	0.342±0.042	3.348±3.590	0.397±0.088
Candidate $Y^{\left(4\right)}$	0.482±0.173	0.244±0.042	2.867±2.579	0.318±0.116
Candidate $Y^{\left(5\right)}$	0.494±0.097	0.342±0.152	4.297±5.017	0.365±0.134
Candidate $Y^{\left(6\right)}$	0.543±0.056	0.488±0.441	2.965±2.888	0.310±0.075
Candidate $Y^{\left(7\right)}$	0.604±0.168	0.293±0.073	3.020±2.938	0.360±0.048
Candidate $Y^{\left(8\right)}$	0.433±0.076	0.269±0.112	4.011±4.193	0.392±0.056
Candidate $Y^{\left(9\right)}$	0.446±0.056	0.195±0.112	3.544±3.764	0.392±0.163
Candidate $Y^{\left(10\right)}$	0.494±0.110	0.317±0.112	3.467±3.542	0.340±0.096
Mean waveform ( $Y^{\left(1\right)}$– $Y^{\left(10\right)}$)	0.470±0.056	0.366±0.073	4.171±4.692	0.350±0.130
Final selected output	0.507±0.021	0.415±0.112	2.923±2.766	0.348±0.080
